# Cutaneous Memoranda

**Published:** 1886-04

**Authors:** 


CUTANEOUS MEMORANDA. By Henry G. Piffard, A. M., M. D., Clinical Professor of Dermatology, University of the City of New York. William Wood & Company. 1885.

This is the third edition of this little manual, and fully sustains the reputation which the first two editions have made. Although the classification of the diseases of the skin is out of date, it being a modification of the one proposed by the French writers of the last century, the little work contains much that is valuable on the subject of treatment.

